# Patients’ and providers’ perspectives on the decision to undergo non-urgent egg freezing: a needs assessment

**DOI:** 10.1186/s12905-023-02743-z

**Published:** 2023-11-13

**Authors:** E. Shirin Dason, Leah Drost, Ellen M. Greenblatt, Adena Scheer, Jinglan Han, Tanya Doshi, Claire A. Jones

**Affiliations:** 1 Department of Obstetrics & Gynecology, Temerty Faculty of Medicine, Faculty of Medicine, University of Toronto, 12th floor, 123 Edward St Toronto, Ontario, M5G 1E2 Canada; 2 Department of Obstetrics and Gynecology, Mount Sinai Hospital, Sinai Health System, Toronto, Ontario M5G 1X5 Canada; 3 Department of General Surgery, St. Michaels Hospital, Unity Health Network, Toronto, Ontario M5B 1W8 Canada; 4 Department of Obstetrics & Gynecology, University of Alberta, Edmonton, Alberta Canada

**Keywords:** Age-related fertility decline, Counselling, Delayed childbearing, Elective egg freezing, Qualitative research

## Abstract

**Background:**

Previous research has demonstrated that patients have difficulty with the decision to undergo non-urgent egg freezing (EF). This study aimed to investigate the decisional difficulties and possible decisional support mechanisms for patients considering EF, and for their providers.

**Methods:**

This qualitative study involved a needs assessment via individual interviews. Participants included patients considering EF at one academic fertility clinic and providers from across Canada who counsel patients considering EF. 25 participants were included (13 providers and 12 patients). The interview guide was developed according to the Ottawa Decision Support Framework. Interviews were transcribed, and transcripts analyzed for themes and concepts using NVIVO 12.

**Findings:**

Multiple factors contributing to decisional difficulty were identified, including: (1) multiple reproductive options available with differing views from patients/providers regarding their importance; (2) a decision typically made under the pressure of reproductive aging; (3) uncertainty surrounding the technology/inadequate outcome data; (4) the financial burden of EF; (5) inherent uncertainty relating to potential decision regret; and (6) differing perceptions between patients/providers regarding the role providers should play in the decision. Additionally, potential sources of decisional support were identified, including provision of basic information before and/or during initial consultation, followed by an opportunity during or after initial consultation for clarifying information and helping with value judgements. Individualized counselling based on patient values, adequate follow-up, psychosocial counselling, and peer support were also emphasized.

**Conclusions:**

More decisional support for women considering EF is needed. Suggestions include a patient decision aid in conjunction with modified healthcare provider counselling, support and follow up.

**Supplementary Information:**

The online version contains supplementary material available at 10.1186/s12905-023-02743-z.

## Background

Non-urgent egg freezing has become increasingly common in Canada, reflecting the trends in delayed childbearing. The average age at which women have their first child in Canada has increased from 23.7 in 1970 to 28.5 in 2011. Strikingly, more than half of all births now occur in women age 30 and older [1]. With an increased recognition of age-related fertility decline and advancements in freezing techniques, there has been a rise in interest in proactive non-urgent oocyte cryopreservation (i.e. egg freezing without an oncological or other medical indication).

Non-urgent egg freezing (EF) has become more widely available within the last five years in Canada, however, there are significant costs associated with it. Costs for an EF cycle in Canada range from $7000—$10 000 for procedural costs, plus $3000-$8000 for medications and $500 in annual storage fees and may vary depending on facility. Inseminating the eggs in the future and transferring embryos has additional costs of $1500- $5000. While it is possible to use Ontario provincial funding for the subsequent fertilization of eggs and embryo transfer, initial EF cycle costs, medication costs, annual storage fees and preimplantation genetic testing are not including in the funding model. In addition to the significant cost of EF, the time commitment and physical experience and risks can be daunting for patients. The process of priming, controlled ovarian stimulation with injectable medications, cycle monitoring with transvaginal ultrasound and bloodwork, and egg retrieval procedure can take 2–8 weeks to complete, with an inherent biological unpredictability in scheduling. Alternative options to EF that patients may consider include, but are not limited to: embryo freezing with donor or partner sperm, trying to conceive now or later with donor or partner sperm, use of donor egg, surrogacy, and adoption.

There have been several rich qualitative studies internationally that have examined the experiences and motivations of women seeking EF [2–8]. Some of these motivations include the desire to disentangle partnership from parenthood, a refusal of social norms, the need for a “back-up plan”, and the idea of taking control of one’s own fertility and future. In addition, there has been a call for individual clinics to develop patient-centred EF protocols/best practices based on qualitative data [7]. Several international guidelines about EF stress the importance of a full discussion of alternatives, risks of EF and prediction of chance of successfully achieving a pregnancy [1, 9, 10]. However, the details of this discussion and how patients and their providers should be supported in this decision have yet to be fully explored in the current literature.

Shared decision-making in which the health care practitioner provides information and a patient shares their values based on their personal beliefs has been identified to be a helpful model of care in obstetrics and gynecology [11]. Assessing the need for decisional support in shared decision-making is the first step to improving decisional quality. Prior studies have explored the motivations and considerations patients face in pursuing EF, and have described various challenges and complexities in their experiences including necessary consideration of many personal values [2, 3]. Furthermore, decisional regret after EF has been demonstrated in a prior study [12]. Prior studies have also suggested that providers may have difficulty supporting patients in pursuing EF [13], or may not feel fully comfortable counselling patients around EF [14, 15]. Thus, the literature supports the identification of decisional supports for this complex shared decision.

A patient decision aid which includes the available options, benefits and harms of each option, probabilities of benefits and risks, and value clarification exercise developed according to current evidence is a healthcare tool which has been increasingly adapted to various clinical problems to support shared-decision making for both providers and patients [11]. In a systematic review, patient decision aids contributed to improved decisional quality via (1) increased patient knowledge, (2) accuracy of risk perception and (3) congruency of care choice with patient values [16]. They also improved the decision-making process by decreasing decisional conflict and emphasizing a less passive patient role [16]. The Ottawa Decision Support Framework (ODSF) was developed specifically for difficult healthcare decisions to assist researchers and providers in assessing patients’ decisional needs/providing decisional support. It offers a standardized way to investigate decisional needs in order to inform the development of a patient decision aid [17]. The ODSF maintains that decision aids which address decisional needs ultimately improve decision quality, equipping patients with improved knowledge, realistic outcome expectations, and values-based decisions [17].

This qualitative study aimed to assess the decisional needs of both patients and providers according to the principles of the ODSF. Our goals were to (1) outline the factors that contribute to decisional complexity, and (2) explore possible decisional supports.

## Materials and methods

### Recruitment and data collection

Data collection involved individual interviews using a semi-structured interview guide based on the ODSF [17]. Interviews were conducted using two populations selected by purposive and convenience sampling: [1] English-speaking patients over 18 attending an academic fertility clinic, Mount Sinai Fertility, in Toronto, Ontario who were considering non-urgent (i.e. non-medical) EF as a reproductive option, and [2] providers (reproductive endocrinology and infertility [REI] physicians, nurse practitioners, reproductive counsellors) across Canada who participate in counselling women about EF as a reproductive option. Patients considering EF for medical reasons were excluded as their motivations for EF and decision-making process would likely differ in important ways from those seeking non-urgent or elective EF. Recruitment tools included bulletin boards at the clinic as well as in-clinic conversations with an attending physician or research assistant. Providers were recruited via email invitation. Sampling was emergent and guided by thematic saturation [18].

Interviews were conducted from November 2018- September 2020 either in person (*n* = 2) or over telephone (*n* = 23) by the investigators E.S.D., L.D. and J.H as well as by research assistant, M.S. Modality was determined by participant preference. Standardized demographic information for participants was collected.

### Interview guides

Two interview guides were developed based on the ODSF framework. The guides were adapted for both populations (patients and providers). The interview guides (Appendix 1) consisted of a mix of open-ended and closed questions regarding the options available to patients, factors that impact the decision-making process and the role of decision support. The guide was considered fluid and interviews were explored according to concepts brought up by participants. Early interviews informed the interview guide, which was modified accordingly in later interviews in an effort to explore new concepts more fully (i.e. uncertainty about EF technology, religious beliefs impacting choice to proceed and the use of EF as a back-up plan). Interviews were recorded and transcribed verbatim by a study investigator. Transcriptions were checked for accuracy by a second study investigator. Concepts of qualitative research such as reflexivity of the researcher, emergent findings, and ongoing analysis were all integrated into data collection and analysis.

### Data analysis

Interviews were subjected to thematic analysis and involved the development of codes, comparison of concepts, and development of theories [18, 19]. Thematic analysis of interview content was reported in a separate paper [20]. For the current paper’s analysis, using the ODSF as a guide, codes were identified and compared to concepts defined by the ODSF around decisional needs, of which the broad categories include: decisional conflict/uncertainty; inadequate knowledge; unrealistic expectations; unclear values; inadequate support/resources; complex decision characteristics; and personal/clinical needs [17]. In keeping with qualitative methodology, data analysis occurred in conjunction with data collection in an iterative process. Interviews were conducted until data saturation was reached. Interviews were independently coded using NVIVO 12 by two study investigators, L.D. and E.S.D. An audit trail of memos and coding was kept to ensure reliability of data. Similarities and discrepancies in coding, concepts and themes were then discussed by L.D., E.S.D., C.J., E.G. and T.H to come to a conclusion.

### Ethics approval

Approval from the Mount Sinai Hospital Research Ethics Board was obtained prior to initiation (study# 17–0001-E). Informed consent was obtained prior to, and throughout the interviews. All methods were performed in accordance with the Declaration of Helsinki.

## Results

Our goal in the current paper was to use the ODSF as a structured guide to (1) identify factors that contribute to decisional difficulty for patients and providers, and (2) identify possible ways to provide decisional support. Thematic analysis of interview content is reported separately [20].

### Demographics

Participant demographics are outlined in Table 1. The current paper is a further analysis of existing interviews reported on previously [20], and no new interviews were conducted for the purposes of the current paper.

**Table 1:** Demographics

Demographic	n (%)
**PATIENTS**	***N* = 12**
***Age (years)***	
30–35	3 (25.0%)
35–39	8 (66.7%)
Not asked	1 (8.3%)
***Education***	
University/college undergraduate	5 (41.6%)
University graduate	4 (33.3%)
Medical degree	1 (8.3%)
Medical and graduate degree	1 (8.3%)
PhD	1 (8.3%)
**PROVIDERS**	**N = 13**
***Years in practice***	
< 5	4 (30.8%)
5–10	4 (30.8%)
10–20	5 (38.5%)
***Gender***	
Female	7 (53.8%)
Male	6 (46.2%)
***Practice role, discipline***	
Physician, GREI	9 (69.2%)
Psychologist/psychotherapist, Fertility	2 (15.4%)
Nurse practitioner, Fertility	1 (7.7%)
Social worker, Fertility	1 (7.7%)
***Location***	
Ontario	6 (46.2%)
Nova Scotia	3 (23.1%)
Alberta	2 (15.4%)
British Columbia	2 (15.4%)

#### Patients

Twenty-three patients were approached for the study, and 14 were interviewed (60.9%). Two interviewed patients were ultimately excluded from analysis as they were undergoing EF for medical indications. Most patients (20/23) who pursued a consultation for EF did end up undergoing EF. One patient chose to undergo EF at another fertility clinic. Only one patient chose embryo freezing only and one patient underwent both embryo freezing and EF.

#### Providers

Twenty-eight providers were approached for the study from across Canada, and 13 were interviewed (46.4%). Most providers were REI physicians, and years in practice varied.

### Factors contributing to decisional difficulty

A number of factors were found, throughout interviews with patients and providers, to contribute to difficulty in decision-making around EF (Table 2). Factors are explored below.

**Table 2:** Factors contributing to decisional difficulty and corresponding ODSF concepts

Factor identified	Corresponding ODSF concept(s) [17]	Selected quotes
Multiple reproductive options with different views regarding importance	Complex decision characteristic: - Difficult decision type (multiple options, outcomes are valued differently by affected population)	*The obvious options are to freeze or not freeze. Then I also considered whether to have a child now [but] I have to rule [that] out right away. I’m still in school, I have no income. I have no partner, so I mean that’s sort of a very theoretical option. That was never really an option.* [Patient 5, age 35–39] *The main options would be expectant management, so wait and see if they meet somebody and conceive naturally, versus proceeding with parenthood right away, for example using donor sperm, versus preserving their fertility with egg freezing, and probably the fourth option, least commonly used, would be to use the eggs and donor sperm to create embryos and freeze embryos.* [Provider 8, REI]
Decision made under the pressure of reproductive aging	Complex decision characteristics: - Difficult decision timing (decision needs to be made soon)	*I understand it’s not gonna help me, as a woman, with letting time bypass. Cause age is the one thing that I have to fight against, you know?* [Patient 2, age 35–39] *I’ll be 35 soon and then I keep hearing that this has to be done by 35 and after that it’s going to be a big impact on your reserve so I would say I was a little bit stressed there.* [Patient 10, age 30–34]
Uncertainty surrounding technology itself	Complex decision characteristics: - Difficult decision type (outcomes are valued differently by affected population)	*So after all this money and all this time and all this worry, all these anxieties, you know, you don’t end up getting a lot of eggs from you and then even if they do, down the line… none of it might work, right? None of it sticks and then all of this would go up in a puff of smoke right?* [Patient 8, age 35–39] *I’d say that the evidence for it, I would say that it’s poor. We’re mostly basing it on algorithms extrapolating it from studies that are from the infertile population. And so there’s still quite a bit of uncertainty as to how successful this treatment really is, and the number of women who actually end up coming back to use their egg is, to date, quite small. So it’s a very new technology I would say* [Provider 3, REI]
Financial burden of EF	Inadequate support & resources: - Inadequate financial assistance (lack access to financial assistance to make/implement decision)	*I think the challenge will always be the cost and how to allocate those funds and hoping – and again it’s we don’t know until we retrieve them, right? So it’s the cost of taking them out, whatever the medication is going to cost at the same time… For myself, I’m only one person. I don’t have a double income. So something like this is something that I’ve been saving for forever, you know.* [Patient 4, age unknown] *It’s not a guarantee as you know, egg freezing. So from that point of view, I think it’s difficult for me to step forward and counsel a patient, ‘Yes, you should definitely do this regardless of the cost’ cause some people may have not a high-paying job, or doing this for instance they have to sacrifice a lot or borrow a lot of money, and I don’t know how important it is for them to have children.* [Provider 2, REI]
Uncertainty about options, EF process, and outcomes	Decisional conflict: - Unsure about what to choose - Worried what could go wrong if they made a choice - Questioned what was important to them - Wavered between choices they made - Constantly thinking about options	*But I still feel very unsure about it. In the sense that emotionally I feel unsure, I’m quite sure about the decision, but emotionally I still feel very hesitant.* [Patient 5, age 35–39] *I guess sort of the unknown around side effects, unknown what time will bring you [makes the decision difficult], like if it’s absolutely necessary at this time. What if you do wait six months from now, will your situation change? So having to make a decision within the face of unknown things.* [Patient 6, age 30–34]
Differing perceptions of decisional roles	Inadequate support & resources: - Feeling unsupported in decision making Inadequate perceptions: others’ views/practices: - Conflicting recommendations from others Difficult decisional roles Classification of preferred roles in decision making: - Other, active or involved - Patient-led - Shared Manifestation of difficult decisional role: - Mismatch between one’s preferred role and actual role in decision-making - Difficulty deliberating with practitioner	*Sometimes I like to hear medical professionals’ own opinion as opposed to like not just being diplomatic, as a patient. I appreciate that too, giving me all the cost and being transparent… [but I want to know] if I were to do this—freeze my eggs and then freeze the embryos, this is something you would recommend for your daughter?… I'm just hoping that a doctor would be ethical enough to be honest with you. Like okay, well truth be told it's probably a waste of your money if you do it twice, right? Or if you have the money, why the hell not, because you're just only giving yourself more chances right? Like that type of advice.* [Patient 8, age 35–39] *I think like with anything in medicine, but in particular with infertility, I think my role is just to inform patients, try to give them an understanding of what the treatment entails, what the costs are, and… how are we determining success, and just allowing the patient to make a decision on their own. So I actually don’t tell patients what to do, I just provide them with the information and let them decide whether they feel like it’s in their best interest or not.* [Provider 3, REI]
Proposed decisional support	Personal and clinical needs: - Clinical – special needs considering health status - Personal – need for tailored information according to age - Clinical – need for tailored information according to diagnosis or its duration	*I think more information [would have helped make the decision]. I think if the clinic would have offered it a lot sooner… You know, just to be better informed, better educated on the entire process.* [Patient 4, age unknown] *I think those three are critical. A, how many eggs you’d anticipate based on their ovarian reserve testing. B, what-what the live birth rate of those eggs would be. And C, what you’d expect the natural decline in fertility would be trying at home. I think those are-if you can personalize those and get them to patients’ hands, either at or after the consult, that would be good.* [Provider 7, REI]

*Abbreviations*: *EF* non-urgent egg freezing, *ODSF* Ottawa Decision Support Framework, *REI* reproductive endocrinology and infertility specialist

#### Multiple reproductive options with different views regarding importance

**Fig. 1:**
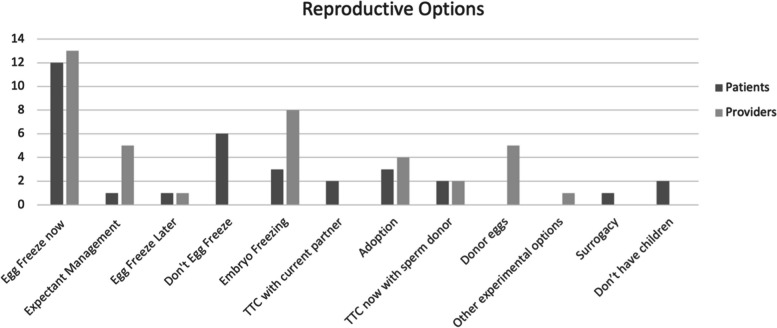
Reproductive options considered by patients versus providers

Reproductive options considered by patients and providers are outlined in Fig. 1. Interestingly, patients primarily saw their options as “egg freeze” or “do not egg freeze”. Other options for childbearing now or in the future, such as embryo freezing with donor sperm, donor egg in the future, or adoption were, for most patients, not considered. This was because of the significant impact of the value placed on reproductive autonomy. Patients ultimately were only considering options that allowed them to conceive with a partner they met in the future. They were looking at EF as true fertility preservation, rather than ensuring they would have a child or a live birth in the future by any means. In keeping with this, when asked to discuss any options for current/future childbearing patients had considered, only 2/12 patients considered “embryo freezing with donor sperm” and only 3/12 patients considered adoption. Patients were highly motivated to pursue EF now to allow for potential pregnancy with their own oocytes in the future, and had not given consideration to alternatives such as conceiving now with donor sperm or conceiving in the future with the use of donor eggs since these were not in keeping with their current life values. As one patient described when asked about options considered, “*The obvious options are to freeze or not freeze. Then I also considered whether to have a child now [but] I have to rule [that] out right away. I’m still in school, I have no income. I have no partner, so I mean that’s sort of a very theoretical option. That was never really an option”* [Patient 5, age 35–39]. In contrast, providers counselled patients on reproductive options that could be utilized throughout their life or were more likely to result in a live birth including “embryo freezing with donor sperm” (8/13) or “donor eggs in the future” (5/13). This discordance highlights a disconnect between what providers think patients will find important to consider, versus what patients realistically view as pertinent to their individual situations.

#### Decision made under the pressure of reproductive aging

Patients needed to make this decision under the pressure of reproductive aging, and this contributed to the inherent difficulty of the decision. In discussing factors that made the decision difficult, one patient shared about the time pressure they felt: “*I’ll be 35 soon and then I keep hearing that this has to be done by 35 and after that it’s going to be a big impact on your reserve so I would say I was a little bit stressed there*” [Patient 10, age 30–34]. Decision timing also impacted the recruitment of patients for this study. Although recruitment was open to any patient who had considered EF, regardless of whether or not they ultimately chose to proceed with it, only patients who chose EF volunteered to participate in the interview. Our observation was that women presenting for EF consultation were not ready to have a child at that time and were therefore only considering options that would allow them to delay this decision and maintain reproductive autonomy, mainly EF now as opposed to conception with donor gametes or embryo freezing.

#### Uncertainty surrounding technology itself

EF was viewed as a newer technology that still remains to be well-established within society. Participants (providers and patients) cited initial testing results, the elements of attrition at each step, and the role of future unknowns – such as future sperm quality or whether the eggs would ever be successfully used – as contributing factors to the inherent uncertainty of the decision. In addition, participants felt that there was inadequate evidence for outcomes in this population. One provider shared, “*I’d say that the evidence for it, I would say that it’s poor. We’re mostly basing it on algorithms extrapolating it from studies that are from the infertile population. And so there’s still quite a bit of uncertainty as to how successful this treatment really is, and the number of women who actually end up coming back to use their egg is, to date, quite small. So it’s a very new technology I would say*” [Provider 3, REI]. Reproductive counsellors also brought up specific considerations about what happens to the genetic material in the event it is not used in the future and the cultural and religious acceptance of children produced via assisted reproductive technology (ART).

#### Financial burden of EF

These decisions carried both a financial and emotional burden. Strikingly, all patients and practitioners brought up the financial burden associated with the decision of whether or not to undergo EF. Most patients felt that the cost of EF was a significant barrier. Costs listed included those associated with consultations, fertility testing, procedures, time off work, and medications. Participants did acknowledge that if a patient was motivated to freeze their eggs, they would find ways to secure the finances, either through personal savings or a loan from family or friends. Several providers shared that the significant financial burden associated with EF negatively impacted their ability to support their patients in their decision-making. Providers felt uncomfortable counselling patients around the value judgement patients needed to make because of the significant cost of EF. As one provider shared, “…*it’s not a guarantee as you know, egg freezing. So from that point of view, I think um it’s difficult for me to step forward and counsel a patient, ‘Yes, you should definitely do this regardless of the cost’ cause some people may have not a high-paying job, or doing this for instance they have to sacrifice a lot or borrow a lot of money, and I don’t know how important it is for them to have children*” [Provider 2, REI].

#### Uncertainty about options, EF process, and outcomes

Patients generally were unsure about which option to choose (i.e. to freeze or not to freeze eggs), and were worried about what could go wrong if they made a choice. In general, patients wanted to delay the decision but, as above, felt the time pressure of reproductive aging to make a decision. They questioned what was important to them and often had to confront personal values to make the decision. In general, when making the decision of whether or not to undergo EF, patients felt upset and physically stressed, wavered between the choices they faced, and constantly thought about their options. Interviews also identified uncertainty in deliberation amongst the multiple options, financial and emotional investment, potential for decisional regret and the physical risk of intervention and medication. One patient described, “*But I still feel very unsure about it. In the sense that emotionally I feel unsure, I’m quite sure about the decision, but emotionally I still feel very hesitant*” [Patient 5, age 35–39].

#### Differing perceptions of decisional roles

Although all participants agreed that this was ultimately a patient-led decision, there was a mismatch in perceived decisional roles. While 5/12 patients looked to providers to share the decision with them, only 2/13 providers actually felt that this was their role. Most REI specialists viewed their primary role as providing information and support to patients and specifically felt uncomfortable helping patients with their value judgements. REI specialists tended to focus on their role as helping with only the comprehension of medical knowledge, the results of initial fertility testing, and the sharing of adequate statistics. They felt that they were responsible for being realistic about the probability of a live birth and emphasizing that EF does not guarantee a genetically-related child in the future. Many providers brought up that they wished they could provide a personal risk calculation to patients based on their age and test results to give them a realistic picture of their potential outcomes. Providers struggled with the inadequacy of current evidence about outcomes in this populations.

Patients emphasized the importance of provider rapport, and in contrast to what providers saw as their role, they stressed need for practitioner support in weighing one’s personal values. One patient shared, “*Sometimes I like to hear medical professionals’ own opinion as opposed to like not just being diplomatic, as a patient. I appreciate that too, giving me all the cost and being transparent… [but I want to know] if I were to do this—freeze my eggs and then freeze the embryos, this is something you would recommend for your daughter?… I'm just hoping that a doctor would be ethical enough to be honest with you. Like okay, well truth be told it's probably a waste of your money if you do it twice, right? Or if you have the money, why the hell not, because you're just only giving yourself more chances right? Like that type of advice*” [Patient 8, age 35–39]. In contrast, providers felt uncomfortable participating in these conversations. One provider shared, *“I think like with anything in medicine, but in particular with infertility, I think my role is just to inform patients, try to give them an understanding of what the treatment entails, what the costs are, and… how are we determining success, and just allowing the patient to make a decision on their own. So I actually don’t tell patients what to do, I just provide them with the information and let them decide whether they feel like it’s in their best interest or not”* [Provider 3, REI].

Both providers and patients felt that there was too much information to share with patients in a short amount of time. Both patients and providers felt limited by the available time during a single consultation and the lack of planned follow-up. Interestingly, patients discussed a desire for more positive messaging. One patient shared, “*I think it needs to be geared towards a little bit more of the positive reason why we're doing all this stuff, rather than it's not going to work; why should we do it? Like I don't want to hear that, you know. Cause it works every day, so I'd like to see a little bit more of that. And I think that does help with the decision*” [Patient 4, age unknown]. This perspective contrasted to providers’ fear of giving a false sense of hope to patients. One provider shared “*We’re very clear that it is not a sure thing. Some clinics I think falsely reassure people that this will give them a child and that’s not how we talk about it – we say it’s a potential option but there’s no guarantee*” [Provider 1, nurse practitioner].

### Proposed decisional support

Ultimately, two timepoints for decision support to address decisional needs were clearly identified by both populations. Most study participants felt that the first opportunity for decisional support was the provision of basic information either before or during the consultation. This information was felt to be most usefully delivered by written information or a website containing multimedia formats that outlined the options available, the benefits and harms, the probabilities of benefits and harms, and the cost information. Providers specifically thought that having a way to demonstrate ovarian reserve depending on a patient’s age and potential live birth outcome would be a helpful addition to the website.

A second timepoint for support was identified as the time during and after consultation with the physician, which should be more focused on helping patients clarify information, asking questions that are important to their values, and assisting in the judgement amongst values surrounding ART. The reproductive counsellor interviewed described talking the patient through different possible future scenarios and helping them question their beliefs and support systems. REI providers, with the available supports that they have, overall felt uncomfortable participating in these types of discussions. Many patients commented on the need for more decisional support in the form of counselling, further conversations with their provider, a prior patient with experience or group sessions with other patients in similar situations.

## Discussion

This study is the first of its kind to compare and contrast both patient and provider views on the decision of whether or not to undergo EF. In the era of shared decision-making for complex healthcare decisions, elucidating provider perspectives for this decision and how they differed from patients’ views highlighted important considerations for counselling. In addition, our study is the first to assess the views of fertility specialists counseling patients about EF in North America. The results of this study highlight decisional needs that can be addressed clinically and through further research moving forward.

Several previous qualitative studies from various higher income countries have also examined motivations and experiences of women undergoing or considering EF [2–8]. Results from our study support aspects of this previous research, emphasizing the impact of reproductive aging and health/career status, relationship status and desired timeline, financial and economic considerations, technological concerns, and the desire to avoid future regret. Our research has also echoed several additional survey-based and cross-sectional studies, which have identified a lack of knowledge about the options, costs, benefits and risks of EF [21], discussed the need for detailed and individualized information on the procedure and possible outcomes for women considering EEF [22], and recognized the need for greater support in the decision-making process [23]. The results of the present study further demonstrate that despite being provided the basic information about EF from their providers, patients have trouble understanding the applicability of the benefits and risks in concordance with their personal values, and desire this additional support from their providers.

There were several areas in which the inclusion of providers’ perspectives offered interesting and novel insights into the decision-making process. Patients in our study generally felt unsure and uneasy about their decision, and wavered between their perceived options, which were typically to either pursue EF or to not pursue EF. Interestingly, in contrast, a previous qualitative study identified that the patients they interviewed in general felt positively about EF, hopeful about their outcomes, and grateful that it was an option they had [7]. However, despite this difference, common to both our patients as well as the patients in the previous study was the idea that they each preferred their provider to relay information and options in a more positive light, and to avoid excessive negativity. In the present study, by involving providers with experience in counselling women around EF, we were able to demonstrate that providers in general were worried about communicating false positivity to patients and, rather, desired to express the objective facts.

Furthermore, the difficulty of financial constraints discussed by all participants in our study is not a novel finding; many other qualitative studies have demonstrated this finding amongst patients as well. Previous studies have found that patients generally feel EF is a good investment and a responsible way to spend their money, and is worth the avoidance of potential future regret [6, 8]. Similarly, in the present study, participants emphasized that if a patient was motivated enough to pursue EF, they would find a way to secure the necessary funding. However, providers interviewed in our study indicated that due to the significant financial burden of EF, they were even more hesitant to engage in shared decision-making as they did not want to present EF as a solid option for patients if they truly could not afford it, perhaps highlighting a degree of paternalism. These findings of incongruency between patients and providers represent potential significant barriers to shared decision-making in the context of EF, and highlight the importance of developing rapport and a strong therapeutic relationship early on in the patient encounter, as well as having discussions around patients’ values and future goals.

The lack of adequate outcome data for EF, an important decisional need, was especially expressed by providers, with many sharing that they wished for a personal risk calculation that they could provide patients about specific live birth outcomes. While this type of personal risk calculation is available in the literature, it exists as predictive models based on outcomes derived from embryo use in an infertile population [24]. As mentioned in previous studies, the population accessing EF for fertility preservation may be very different from other populations, such as those accessing IVF for infertility [7]. These existing predictive tools could be adapted into clinical practice now, but as these populations likely have very different decisional needs, our research does call for the need of clinic-specific outcome data in the context of EF for fertility preservation, perhaps shared amongst clinics, to better support both patients and providers in shared decision-making.

Our study, by including providers involved in this decision and focusing on the idea of shared decision-making, specifically identified factors that could assist in the implementation and improvement of this practice. Basic information can be provided to patients in the form of written material, online material or videos prior to consultations to preserve valuable clinical time for working through the decision itself. Furthermore, many patients in our study expressed a desire for providers to play a more active role in the decision. While this is ultimately a patient-led decision, patients identified the need for help deciding where they should place importance. Patients desired for providers to help them examine the values important to them, rather than to simply tell them the necessary facts and information. Many providers, in contrast, indicated that they felt uncomfortable participating in these types of discussions with patients, as they felt it was up to the patients to perform this type of value judgement. The separate finding that patients and providers differ in what they perceive as available reproductive options for patients furthermore indicates that these types of value-based discussions may not be readily occurring; our finding of patients primarily only considering EF as opposed to other childbearing methods such as adoption or sperm donation has also been identified in a previous study [3]. Our results highlight a disconnect between patient expectations and what providers feel they are capable of providing; both patients and providers need more support in these discussions.

Several specific decisional supports were suggested. First, some providers may appreciate guidance in how to participate in a discussion around EF decision-making, particularly in how to engage in a values-based discussion. As mentioned, some patient suggestions on what they looked for from their providers included framing the probabilities in a different light, similar to how a provider might focus on the positive aspect of a prognosis probability when someone is faced with an illness. For example, phrasing could be adjusted to “There is a 50% chance you will have a child” from “There is a 50% chance you will not have a child”. Specific questions that patients found useful to be asked included “What is the value of your eggs?” (i.e. is the value of your eggs worth the cost of freezing them), or “How would you feel if you were never a parent?” and “How old do you want to be relative to your child?”. Other suggestions for improved shared decision-making, which support prior studies, include longer consultation times and ensured follow-ups in 3–6 months’ time [25] in order to facilitate rapport and in-depth conversation. For those providers who feel this is out of their scope of practice, referral to a reproductive counsellor may be an appropriate solution. A patient decision aid to be used in conjunction with counselling which can incorporate a values clarification exercise would also be an attractive option. Ideally, a centralized source adopted by multiple clinics would mitigate the concern of financial motivation of private clinics and provide a sense of regulation to patients.

The main strength of this study was the incorporation of both provider and patients views, and it is the first of its kind to do so. Providers were recruited across Canada and included multiple disciplines. In addition, we used a standardized framework to assess decisional needs; the analysis was accomplished using the recently published ODSF decisional needs assessment. Limitations of this study included patient recruitment from a single academic-affiliated, urban fertility clinic which may not be representative of the whole population of women seeking EF. Patients were also all highly educated and English-speaking, which may not be reflective. In addition, all participants had chosen to undergo EF. Though this is a limitation, it was clear from the interviews that women presenting for consultation are highly motivated to pursue EF over another reproductive option. Future studies could consider recruiting patients more broadly from social media or primary care practice to address this limitation. Finally, patients were not interviewed again at a later date limiting exploration of decisional regret and future consideration of other options.

## Conclusions

In conclusion, our study has explored the decisional needs of patients and providers in considering EF, and identified factors that contribute to decisional difficulty. Additionally, we have identified that decisional support may be beneficial. This study is the first of its kind to include the perspectives of providers in this setting, which lent an additional level of insight into the decision-making process. Specific factors that can support this decision and aid in shared decision-making between patients and providers include the provision of background information prior or during consultations, taking time to review patient values (i.e. where they should place importance based on their personal values) in the context of the complex medical information, consideration of a referral to a reproductive counsellor, and ensuring adequate follow-up. Some of these elements may be addressed by a patient decision aid. Whether the interventions subsequently improve decisional outcomes would need to be assessed in future studies.

### Supplementary Information

**Additional file 1: Appendix 1. **Interview Guides.

## Data Availability

The data underlying this article cannot be shared publicly due to the privacy of the individuals that participated in the study. The data will be shared on reasonable request to the corresponding author.

## Abbreviation

EF: Egg freezing
ODSF: Ottawa Decision Support Framework
